# Genital Schistosomiasis in European Women

**DOI:** 10.5402/2011/242140

**Published:** 2011-06-09

**Authors:** Xavier Catteau, Anass Fakhri, Valérie Albert, Brahima Doukoure, Jean-Christophe Noël

**Affiliations:** ^1^Department of Pathology, Erasme Hospital, Université Libre de Bruxelles, Route de Lennik, 808, 1070 Brussels, Belgium; ^2^Department of Gynaecology, Erasme Hospital, Université Libre de Bruxelles, Route de Lennik, 808, 1070 Brussels, Belgium

## Abstract

Female genital schistosomiasis (FGS) is an isolated chronic form of schistosomiasis. Although most infections occur in residents of endemic areas, it has been clearly documented that brief freshwater exposure is sufficient to establish infection; thus, travellers may also be infected. The clinical manifestations of FGS are nonspecific, and lesions may mimic any neoplastic or infectious process in the female genital tract. It is important to take a careful history and physical examination, making sure to consider travel history in endemic areas. The diagnosis is confirmed by microscopy with egg identification or by serology. The standard of care for treatment is a single dose of oral praziquantel which avoids complications and substantial morbidity. Herein, we report a rare and original case of FGS in a European woman.

## 1. Introduction

Schistosomiasis was first described by Theodor Bilharz in 1851 [1]. It is estimated that more than 200 million people worldwide have schistosomiasis and that the infection is responsible for more than 200,000 deaths annually [2]. Environmental changes that result from the development of water resources and the growth and migration of populations can facilitate the spread of schistosomiasis [1]. Although most infections occur in residents of endemic areas, it has been clearly documented that brief freshwater exposure is sufficient to establish infection; thus, travellers may also be infected [3]. There is no accurate information about infection rates among returned travellers and immigrants because it is a rare disease in western countries [1]. Herein, we report a rare case of female genital schistosomiasis (FGS) in a European woman.

## 2. Case Report

**Figure 1:**
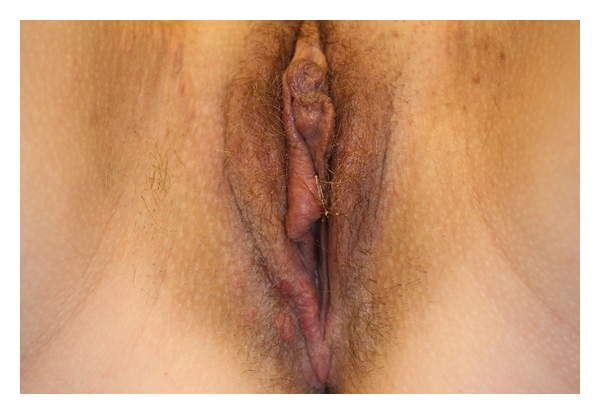
Clinically, we observed a swelling of the right labium minus.

A European 20-year-old woman was referred from our mucous clinic with a history of a vulvar mass evolving for three weeks. Her past medical and surgical history was unremarkable. This mass was painful and was increased in size. The patient went to Senegal and Mali three months ago, and she had a history of swimming in fresh water lakes in these countries. Clinically, we observed a swelling of the right labium minus (Figure 1). This mass was painful with a soft consistency. No other cutaneous or genital lesion was found. She had no fever, urticarial rash, angioedema, dry cough, or wheeze. No hematuria and no dysuria were reported. Hepatomegaly and splenomegaly were absent. Following laboratory parameters were within normal limits: complete blood cell count, serum electrolyte profile, and liver function tests. No antischistosomal antibodies were found in the serum, and there was no eosinophilia. Microscopy of urine and stools was negative for ova, cysts, and parasites and did not contain red blood cells or white blood cells. Biopsy of the vulvar lesion was made. 

**Figure 2:**
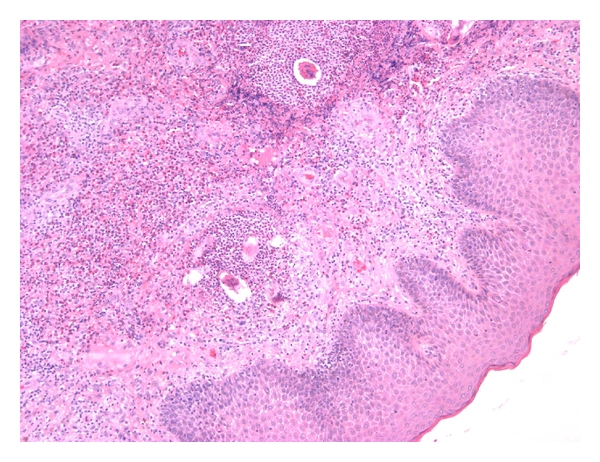
A noncaseating granulomatous reaction to clusters of viable-appearing eggs in the dermis (Haematoxylin-eosin ×100).

**Figure 3:**
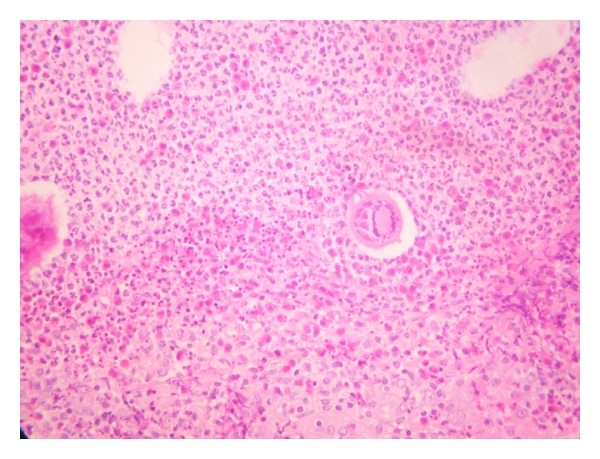
The granuloma is composed of epitheloid histiocytes and a lot of eosinophils and plasma cells (Haematoxylin-eosin ×200).

**Figure 4:**
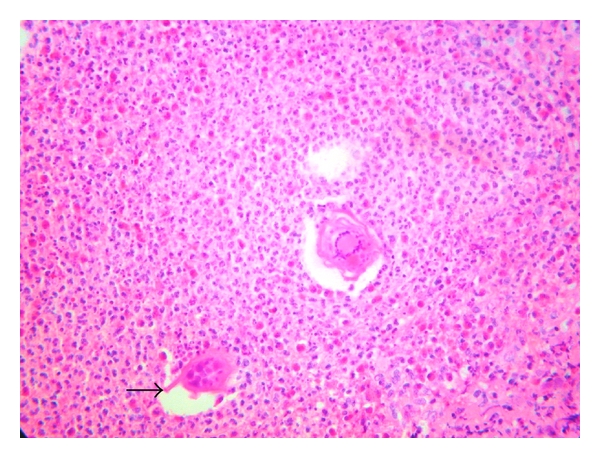
Histology revealed numerous schistosome ova with terminal spines (arrow) characteristic of *Schistosoma haematobium* (Haematoxylin-eosin ×400).

Histological examination at low-power view revealed a noncaseating granulomatous reaction to clusters of viable-appearing eggs in the dermis (Figure 2). The granuloma was composed of epitheloid histiocytes and a lot of eosinophils and plasma cells surrounded by a rim of lymphocytes (Figure 3). The epidermis was moderately acanthotic with hyperkeratosis. We observed foci of spongiosis and exocytosis in front of the infiltrate. At high-power view, histology revealed numerous schistosome ova with terminal spines, characteristic of *Schistosoma haematobium*. The ova contained multiple viable miracidia surrounded by a marked granulomatous inflammatory infiltrate (Figure 4). The diagnosis proposed was FGS. The patient was treated with oral praziquantel at a dose of 2400 mg for one day. She came back to our clinic six months later with disappearance of the vulvar lesion, and no others symptoms were reported.

## 3. Discussion

Schistosomiasis is the infection of humans by trematodes (a class of helminths). Three major species of schistosomes are described: *S. haematobium*, *S. japonicum*, and *S. mansoni* [4]. *S. haematobium* infection is acquired predominantly in North Africa, sub-Saharan Africa, the Middle East, Turkey, and India. The majority of *S. mansoni* infections are found in sub-Saharan Africa. *S. japonicum* infection still occurs in China, Indonesia, and the Philippines [1, 5]. Morphologically, *S. haematobium* ova have a spine in the apical position, whereas the spine of *S. mansoni* ova is on the lateral aspect, and *S. japonicum* ova have no spine [5]. FGS is the isolated chronic form of genital schistosomiasis. No other symptom is reported. *S. haematobium *is the organism most often identified in FGS [1, 4, 6]. In most cases, the lesions are found on the labia majora [6]. Isolated internal genital disease is less frequent [1]. In postmortem studies of the reproductive tract in endemic areas, ova are found in the vulva in 7 to 17% of cases [7].

The life cycle of schistosomiasis is complex and requires both intermediate and definitive hosts. When eggs excreted from infected humans contaminate bodies of fresh water, they hatch to release free-swimming miracidia, which in turn infect snails, the intermediate hosts. The miracidia mature inside the snails to become cercariae, which are released back into the water and can penetrate the skin of humans. Cercariae can survive up to 48 hours in water but are most infectious to humans in the first few hours after release from the snail. Cercariae pass via the lung and the liver into the portal venous and then mature into adult worms and unite. Pairs of worms then migrate to the superior mesenteric veins (in the case of *S. mansoni*), the inferior mesenteric and superior haemorrhoidal veins (in the case of *S. japonicum*), or the vesical plexus and veins draining the ureters and the urinary bladder (in the case of *S. haematobium*). Live schistosome eggs are excreted in the feces (in the case of *S. mansoni* and *S. japonicum*) or urine (in the case of *S. haematobium*). Direct retrograde spread of the adult worms from their usual sites into the venous system supplying vulvar skin leads to deposition of ova in the skin and subsequent formation of genital lesions, as in our patient. The life cycle is completed when the eggs hatch, releasing miracidia that, in turn, infect specific freshwater snails [1, 4, 5]. The adult worms remain in the blood vessels for life and survive for five to seven years and can even persist for up to 30 years [8].

Physiopathologically, 2 to 3 cm adult worms may cause venous obstruction where they reside, but the disease is more commonly caused by the daily deposition of numerous eggs by the female (hundreds to thousands eggs per day, depending on the species), which invades local tissues, where they release toxins and enzymes and provoke a TH-2-mediated immune response [4, 8, 9]. Inflammation and granuloma formation occur around deposited eggs, which can lead to extensive tissue damage (fibrosis and scarring) [4, 10]. Most patients infected with schistosomes of all species are asymptomatic. Acute symptoms tend to be more common in nonimmune individuals, such as travellers, due to a more intense immune response to exposure. By contrast, chronic complications require a higher burden of infection and, thus, are mainly seen in individuals from endemic areas [11]. The clinical picture of genital schistosomiasis and the pathological findings vary according to the organ affected. The clinical manifestations of FGS are nonspecific except the presence of sandy patches (areas of roughened mucosa surrounding egg deposits) on the cervical surface [7, 12]. It can include irregular bleeding, discharge, pelvic pain or tenderness, dyspareunia, and otherwise unexplained infertility. The lesions are polypoid or papillomatous tumorlike lesions on the vaginal wall and vulva. Macroscopically, lesions in FGS may mimic any neoplastic or infectious process (e.g., condyloma lata, syphilis, squamous cell carcinoma) in the female genital tract [1, 4, 13]. Lesions of the vulva and clitoris vary in size and presentation. Progressive and relapsing swelling, painful or painless ulceration, nodular surface, pruritus, and a hypertrophic clitoris with an eroded granular surface have been described. Genital schistosomiasis started with an irritation of the skin, followed by oedema and hyperaemia. Later small nodules developed under the skin and papillomatous lesions appeared forming masses resembling condylomata [14]. In travellers returning from the tropics, due to the unspecific nature of gynaecological findings, delays of more than 24 months in diagnosis have been reported, the lesions being misdiagnosed as, for example, vulval warts [14].

The diagnosis of FGS is first suspected after taking a careful history and physical examination, making sure to consider travel history in endemic areas. It can be confirmed by microscopy with egg identification or by serology [4, 15]. Eggs can be directly visualized in urine or feces, but this test has limited use in the diagnosis of FGS, because FGS may exist independently and without signs of urinary schistosomiasis [4, 14, 16]. Studies in *S. haematobium*-endemic areas have also shown that up to 23% of women may have involvement of the lower reproductive tract even without schistosome ova in the urine [7].

The complications with *S. haematobium* are granulomatous inflammation, ulceration of the vesical and ureteral walls with subsequent fibrosis, functional bladder neck obstruction, hydroureter, hydronephrosis, and calcifications of the urinary tract and bladder. Genital involvement can cause infertility secondary to ovarian fibrosis or tubal occlusion. *S. haematobium* could be a cofactor for the development of cervical cancer and squamous cell carcinoma of the bladder, but a clear link has yet to be established. Finally, FGS may also facilitate the transmission of human immunodeficiency virus [1, 4, 7, 12, 16].

The standard of care for treatment is a single dose of oral praziquantel, a pyrazinoisoquinoline derivative, at a dose of 40 mg/kg [1, 4, 13]. The drug's precise action on adult worms is unknown. Although praziquantel is not considered to be teratogenic or mutagenic, the drug is not recommended in pregnancy or in lactating women [14]. Reexamination of feces or urine one month after treatment is recommended in order to assess efficacy [1]. In our patient, this control is useless because the urinary and feces tests were already negative before the treatment. Meltzer et al. propose to screen and treat asymptomatic travellers with history of freshwater exposure in endemic countries [17].

In conclusion, female genital schistosomiasis is a rare disease in the western world. Our patient illustrates an unusual presentation of schistosomiasis via the localisation of the lesion and the patient's origin. It should be born in mind as a cause of granulomatous inflammation on the genitalia and must enter into the differential diagnosis of symptomatic genital lesions when the clinical history indicates travel in endemic areas. Eggs can be identified by cytologic or histologic methods and appropriate treatment can be initiated, avoiding substantial morbidity.
